# Varying association of nutrient intakes with quality of life in patients receiving different modes of dialysis

**DOI:** 10.3389/fcvm.2024.1407650

**Published:** 2024-05-27

**Authors:** Yadi Guo, Dongling Luo, Li Yin, Xiaoyan Su, Zhimin Yuan, Hui Huang, Jie Chen

**Affiliations:** ^1^ Cardiovascular Department, The Eighth Affiliated Hospital, Sun Yat-sen University, Shenzhen, China; ^2^ Department of Nephrology, Dongguan Tungwah Hospital, Guangdong, China; ^3^ Department of Clinical Nutrition, Sun Yat-Sen Memorial Hospital, Sun Yat-Sen University, Guangzhou, China; ^4^ Department of Radiation Oncology, Sun Yat-sen Memorial Hospital of Sun Yat-sen University, Guangzhou, China

**Keywords:** nutrient, malnutrition, quality of life, hemodialysis, peritoneal dialysis

## Abstract

**Background:**

Nutrients are crucial for dialysis patients, especially elderly patients. Nutrition-related complications in dialysis patients are often closely related to cardiovascular aging. However, we know little about the effect of different nutrients on the commonly used outcome predictor, health-related quality of life (HRQOL). Therefore, this study investigated the associations between different nutrients and HRQOL among dialysis patients.

**Methods:**

A cross-sectional study was conducted on 123 dialysis adults at multiple dialysis centers. The Short Form-36 Health Survey (SF-36) assesses HRQOL. Modified quantitative subjective global assessment (MQSGA) evaluates nutritional status. A 3-day dietary record evaluated nutrient intakes.

**Results:**

Among the 123 participants, 79 received hemodialysis (HD), and 44 were on peritoneal dialysis (PD). Patients with PD had a higher SF-36 score than HD (525 ± 136 vs. 375 ± 179, *P* < 0.001). A negative association between nutrition status and HRQOL was observed in HD (regression coefficient *β* = −17.4, *P* < 0.001) but not in PD (*β* = −12.3, *P* = 0.07). For HD patients, the nutrition status was negatively correlated with intakes of carbohydrates, fiber, selenium, copper, and Manganese (*β* = −0.02, *P* = 0.032; *β* = −0.3, *P* = 0.031; *β* = −0.1, *P* = 0.006; *β* = −2.3, *P* = 0.025; *β* = −1.3, *P* = 0.003, respectively). Their HRQOL was positively associated with calories, fat, niacin, and vitamin E (*β* = 2.19, *P* = 0.035; *β* = 2.4, *P* = 0.043; *β* = 8.5, *P* = 0.044; *β* = 6.9, *P* = 0.017, respectively). Conversely, for patients with PD, only vitamin B2 was found to be adversely correlated with their nutritional status (*β* = −5.2, *P* = 0.037), and increased intakes of vitamin A, vitamin C and fiber (*β* = 0.1, *P* = 0.031; *β* = 0.8, *P* = 0.028; *β* = 15.8, *P* = 0.045, respectively) were associated with a better HRQOL.

**Conclusions:**

The nutritional intake of PD patients and HD patients affects their quality of life differently. Macronutrients significantly impact HRQOL in HD patients, while vitamins have a more substantial impact on PD patients.

## Introduction

Approximately 10% of the population worldwide suffers from chronic kidney diseases, and nearly 3 million are receiving maintenance dialysis (1). Though life-saving, dialysis is a time-consuming and rigorous process that affects patients' lives in almost all aspects, either physically or psychosocially (2). Among maintenance dialysis patients with stage 3–5 CKD, the incidence of malnutrition, such as hyperphosphatemia, protein-energy malnutrition, and water and sodium balance disorders, is as high as 28%–54% (3). These complications are closely related to mitochondrial function and cardiovascular health. Studies have shown that protein-energy malnutrition increases the risk of major cardiovascular events (4), and vitamin C also affects regulating oxidative stress and mitochondrial function (5). Therefore, the nutritional status of dialysis patients requires additional attention.

Dietary intake is paramount to patients with chronic kidney disease (CKD) (6, 7). Inappropriate nutrient intakes might accelerate disease progression, increasing the risk of malnutrition and frailty and negatively impacting patients' quality of life (8). Besides, as with deteriorating renal function, the ability to remove waste products is compromised, and hence, dynamic change of the nutrient recommendations should be considered at different stages of kidney disease (9). Hemodialysis (HD) and Peritoneal dialysis (PD) are technically two types of renal replacement therapy; thus, nutrient loss or requirement might vary between these two modalities. Further, emerging evidence has suggested that the effect of nutrient intake differs in patients treated with different types of dialysis. As shown in our previous studies and other teamwork, the relationships of specific nutrients to nutritional status were distinctive in HD and PD patients (10–12).

Multiple studies have shown that the health-related quality of life (HRQOL) of patients treated with maintenance dialysis is markedly impaired compared with the general population (13, 14). HRQOL is a multidimensional concept usually evaluated by the Short Form-36 Health Survey (SF-36). This subjectively perceived HRQOL has been considered a predictor of more objective outcomes such as prospective hospitalization and mortality (15).

However, whether the effect of nutrient intake differs across different dialytic modes and how they would influence the quality of life in dialysis patients remains understudied. Therefore, the purpose of our study was to (1) test the effects of dialytic modalities and nutritional status on patients' quality of life; (2) determine the relations of different nutrients to nutritional status in patients with HD and PD; (3) most importantly, investigate the associations between different nutrient intakes and quality of life in patients treated with different types of dialysis.

## Materials and methods

### Participants

This cross-sectional study included patients undergoing dialysis at four dialysis centers in South China (Kiang Wu Hospital; the Affiliated Hospital of School of Chinese Medicine, the University of Hong Kong; Tungwah Hospital of Sun Yat-Sen University, Dongguan and Hemodialysis Center, the First Affiliated Hospital of Guangdong Hospital University of Pharmacy, Guangzhou) during January 1, 2012 and September 30, 2019. Participants were randomly selected according to the following criteria: (1) aged above 18 years old, (2) diagnosis of end-stage renal disease (ESRD), (3) receiving maintenance dialysis for at least three months, and (4) volunteer participation. Patients were excluded if they were seriously infected, hospitalized one month before data collection, unable to understand or complete the SF-36 survey and finish the 3-day dietary records, or suffering from active malignancies with shorter than six months of life expectancy. The study protocol conformed to the ethical guidelines of the 1975 Declaration of Helsinki and was approved by the Ethics Committee of Sun Yat-sen University. Written informed consent was obtained from each patient.

### Anthropometric and nutrient intake assessment

Weight, height, waist circumference, mid-upper arm circumference and triceps skin fold thickness were measured using techniques described more detailedly in our previous study (11, 12). Body mass index was calculated as body weight divided by height squared. Dietary intake was recalled for hemodialysis patients over the last hemodialysis treatment day of the week and the two subsequent non-dialysis days. The nutrient intakes were evaluated for peritoneal dialysis patients for three consecutive days. Patients recorded the intake of each food and the number of meals (such as breakfast, lunch, dinner and snacks) per day in a food diary to determine dietary intake. The dietitians then verified the food records and quantified the nutrients via the Minnesota Nutrient Data System software (version 2005; Nutrition Coordinating Center, Minneapolis, Minn, USA) and Dietwin® Nutrition software (version 8.0).

### Nutritional assessment

Nutritional status was performed by a trained nutritionist using the modified quantitative subjective global assessment (MQSGA). MQSGA consists of 7 questions, including weight change during the past six months, dietary intake, gastrointestinal symptoms, functional capacity, and co-morbidities. Physical examination assessed loss of subcutaneous fat, muscle wasting, and nutrition-associated alterations in fluid balance, edema or ascites. Each component was rated on a scale of 1 (normal) to 5 (very severe). The sum of all MQSGA components ranges from 7 to 35. A higher total score reflects a more severe degree of malnutrition. The current study classified nutritional status as normal or malnutrition based on the cutoff value 14. An MQSGA score below 14 was considered normal nutrition, while above 14 was malnutrition (16, 17).

### Health-related quality of life assessment

Health-related quality of life was measured using the SF-36 survey. This survey has been used in various countries on dialysis patients and is valid and reliable (18, 19). The SF-36 survey includes one multi-item scale that assesses eight health concepts: physical functioning (PF), role physical functioning (RP), bodily pain (BP), general health (GH), vitality (VT), social functioning (SF), role emotional functioning (RE), and mental health (MH). Scores were assembled using the Likert method for summated ratings; raw scores were linearly transformed into 0–100 scales, with 0 and 100 assigned to the lowest and highest possible values, respectively; higher scores indicated a better outcome (19). A summary of total SF-36 scoring was calculated for all participants. Health Questionnaires were completed with the aid of study personnel if these participants had difficulty with self-administration.

### Statistic methods

Data were presented as mean ± standard deviation or percentages, as appropriate. Differences between continuous variables were assessed using a student's *t*-test. The chi-square test or Fisher's test compared categorical variables among groups. The relationships between dialysis modality and SF-36 score, MQSGA score and SF-36 score were determined by linear regression models, either in adjusted (age, gender, body mass index, total calorie, protein, carbohydrate, and fat consumption) or non-adjusted models. Additionally, subgroup analyses were performed based on age (< or ≥60 years old), nutritional status (normal nutrition or malnutrition), or different dialytic modality (hemodialysis or peritoneal dialysis).

On analyzing the relationship between nutrient intakes and nutritional status, as well as different facets of patients' HRQOL, univariate linear regression analysis was performed separately in HD and PD subgroups. Since multiple nutrients were analyzed, we tried to find whether there was a moderation or interaction effect between different nutrient intakes on the same aspects of HRQOL. An interaction product was produced in the moderation model, and its role in the regression model was tested (20). A standardized coefficient was used due to inconsistent units of different variables.

The results were reported as regression coefficient (*β*) and 95% confidence interval (95% CI). All *p*-values were 2-sided, and a value at *p* < 0.05 was considered statistically significant. All analyses were performed using Empower(R) (www.empowerstats.com, X&Y Solutions, Inc., Boston, MA) and R (http://www.R-project.org).

## Results

### Participants and characteristics

**Figure 1:**
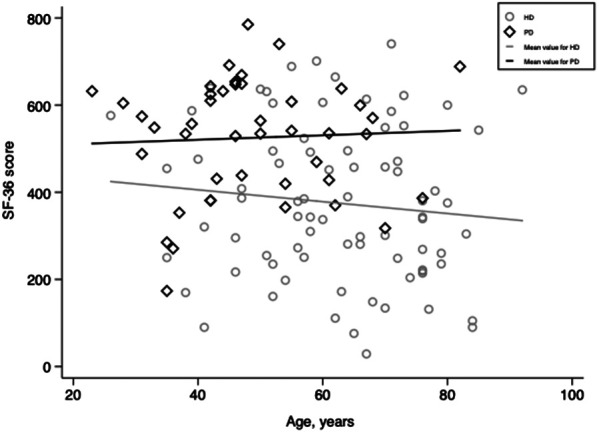
Scatter plots of SF-36 scores for patients receiving hemodialysis and peritoneal dialysis. HD, hemodialysis; PD, peritoneal dialysis.

**Figure 2:**
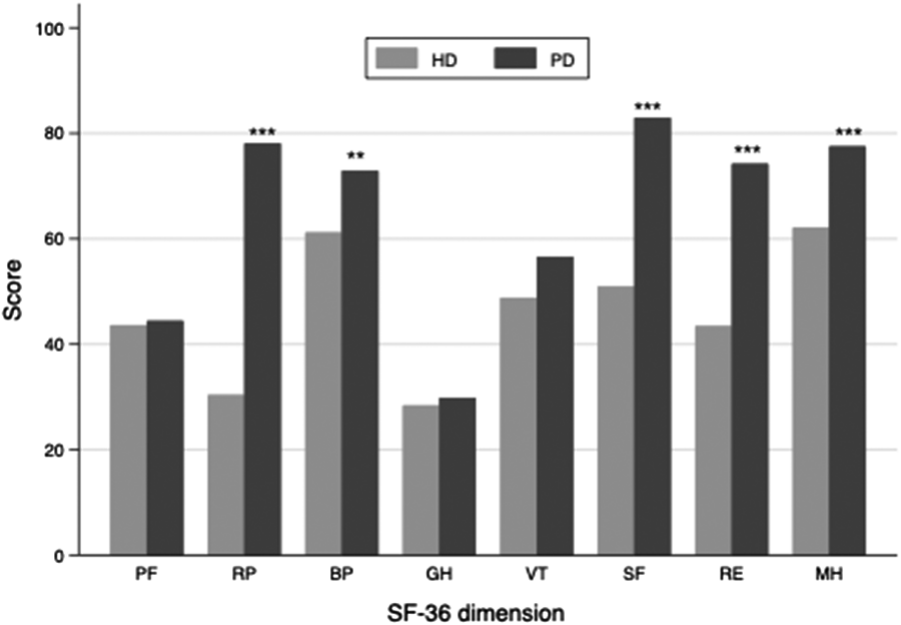
Bar chart of the SF-36 subscale score in hemodialysis and peritoneal dialysis subgroup. The PF, SF, RE and MH subscale scores were significantly higher in peritoneal dialysis than in the hemodialysis subgroup. PF, physical functioning; RP, role-physical functioning; BP, bodily pain; GH, general health; VT, vitality; SF, social functioning; RE, role-emotional functioning; MH, mental health; HD, hemodialysis; PD, peritoneal dialysis. *** indicates a *p* value <0.001, ** indicates a *p* value < 0.05.

One hundred twenty-six adults receiving dialysis for at least three months were included. Three of them refused to complete the SF-36 questionnaires and thus were excluded. Finally, 123 dialysis adults were included in our study. The overall mean age was 58 ± 15, and 66% of participants were men. Among the study population, 79 received hemodialysis (HD), and 44 were on peritoneal dialysis (PD). Overall, patients receiving PD were significantly younger (48 ± 13 vs. 63 ± 14, *p* < 0.001) and had higher SF-36 scores (525 ± 136 vs. 375 ± 179, *p* < 0.001, Figure 1) than patients in the HD subgroup. More specifically, patients with PD perceived better feelings in role-physical functioning, social functioning, role-emotional functioning, and mental health (Figure 2). However, as presented in Table 1, patients in the PD subgroup consumed less of almost all nutrients, except vitamin A and C, than HD patients. Despite this, the body mass index (21.6 ± 3.1 vs. 21.9 ± 4.5 kg/m^2^, *p* = 0.68) and malnutrition rate (38% vs. 32%, *p* = 0.38) did not differ significantly between groups.

**Table 1:** Baseline characteristics of patients receiving hemodialysis (HD) or peritoneal dialysis (PD).

	Total (*n* = 123)	HD (*n* = 79)	PD (*n* = 44)	*P*-value
Age, years	58 ± 15	63 ± 14	48 ± 13	<0.001
Gender, male, *n* (%)	82 (66%)	51 (65%)	31 (70%)	0.50
Body mass index, kg/m^2^	21.7 ± 3.7	21.6 ± 3.1	21.9 ± 4.5	0.68
MAC, cm	26.1 ± 3.7	25.8 ± 4.1	26.5 ± 2.9	0.32
TSF, mm	10.9 ± 6.4	10.7 ± 6.5	11.2 ± 6.3	0.67
MQSGA score	13.0 ± 4.0	13.0 ± 4.4	13.0 ± 3.2	0.96
Malnutrition, *n* (%)	44 (36%)	30 (38%)	14 (32%)	0.38
SF-36 score	429 ± 179	375 ± 178	525 ± 136	<0.001
Dietary intake				
Total calorie, kcal/d	1,753.2 ± 369.9	1,810.7 ± 391.5	1,380.6 ± 269.5	<0.001
Total calorie, kcal/kg/d	30.4 ± 6.3	31.4 ± 6.7	24.2 ± 4.7	<0.001
Protein, g/d	65.7 ± 21.0	74.5 ± 20.1	50.7 ± 12.2	<0.001
Protein, g/kg/d	1.1 ± 0.3	1.3 ± 0.3	0.9 ± 0.2	<0.001
Carbohydrate, g/d	216.1 ± 59.6	233.8 ± 62.1	185.6 ± 40.3	<0.001
Fat, g/d	58.2 ± 17.2	63.9 ± 17.1	48.5 ± 12.4	<0.001
Fiber, g/d	9.2 ± 3.8	9.4 ± 4.3	8.7 ± 2.7	0.31
Cholesterol, mg/d	385.7 ± 245.9	469.2 ± 255.0	242.1 ± 143.7	<0.001
Vitamin A, μg RE/d	602.9 ± 385.3	596.6 ± 412.8	613.7 ± 337.0	0.82
Vitamin B1, mg/d	0.9 ± 0.3	1.0 ± 0.3	0.8 ± 0.2	0.03
Vitamin B2, mg/d	0.8 ± 0.5	1.0 ± 0.6	0.6 ± 0.2	<0.001
Niacin, mg NE/d	14.1 ± 4.9	15.7 ± 4.8	11.3 ± 3.7	<0.001
Vitamin C, mg/d	108.7 ± 71.6	100.5 ± 76.7	122.8 ± 60.1	0.10
Vitamin E, mg/d	20.4 ± 6.3	22.0 ± 7.0	17.5 ± 3.2	<0.001
Calcium, mg/d	409.2 ± 212.2	441.5 ± 244.1	353.5 ± 125.9	0.03
Phosphate, mg/d	833.7 ± 264.6	940.2 ± 258.0	650.5 ± 153.0	<0.001
Potassium, mg/d	1,565.7 ± 523.6	1,707.6 ± 570.1	1,321.7 ± 309.7	<0.001
Sodium, mg/d	706.4 ± 462.1	837.5 ± 505.7	480.7 ± 250.9	<0.001
Magnesium, mg/d	227.3 ± 71.5	244.3 ± 79.0	198.0 ± 43.6	<0.001
Iron, mg/d	15.2 ± 4.7	16.7 ± 5.0	12.6 ± 2.7	<0.001
Zinc, mg/d	9.4 ± 2.9	10.6 ± 2.9	7.4 ± 1.8	<0.001
Selenium, ug/d	43.4 ± 24.3	53.9 ± 24.4	25.2 ± 7.7	<0.001
Copper, mg/d	1.3 ± 0.5	1.5 ± 0.6	1.1 ± 0.3	<0.001
Manganese, mg/d	3.7 ± 1.2	4.1 ± 1.3	3.2 ± 0.7	<0.001

Data represented as mean ± standard deviation and number (%).

MAC, mid upper arm circumference; TSF, triceps skin thickness; MQSGA, modified quantitative subjective global assessment. SF-36, Short Form-36 health survey.

### The effect of dialytic modalities and nutritional status on HRQOL

Linear regression analysis was performed, and a positive relationship between PD and SF-36 was found (*β* = 149.8, *p* < 0.001), taking HD as a reference. This result remained significant after adjusting for potential confounders, including age, gender, body mass index (BMI), total calorie, protein, carbohydrate, and fat consumption (*β* = 193.6, *p* = 0.001). In addition, this association was observed in different age strata or at different nutritional statuses, with a more substantial relation in patients older than 60 years old or with normal nutritional status (Table 2).

**Table 2:** Relationships between dialysis modality, nutrition status (MQSGA score) and SF-36 score.

	Dialysis modality^a^, peritoneal dialysis (*β*, 95% CI)	*P* value	MQSGA score (*β*, 95% CI)	*P* value
Different models				
Non-adjusted	149.8 (89.2, 210.4)	<0.001	−16.2 (−24.0, −8.4)	<0.001
Adjusted I	137.2 (68.0, 206.4)	<0.001	−16.7 (−25.0, −8.4)	<0.001
Adjusted II	193.6 (79.3, 308.0)	0.001	−18.1 (−26.5, −9.6)	<0.001
Age subgroup				
Age < 60 years	129.0 (54.5, 203.5)	0.001	−15.0 (−26.2, −3.7)	0.01
Age>=60 years	150.4 (30.5, 270.2)	0.02	−17.9 (−28.3, −7.5)	0.001
Nutritional status subgroup				
Normal nutrition	89.0 (12.8, 165.2)	0.03	–	–
Malnutrition	198.8 (102.0, 295.6)	<0.001	–	–
Dialysis modality subgroup				
Hemodialysis	–	–	−17.4 (−26.2, −8.7)	<0.001
Peritoneal dialysis	–	–	−12.3 (−25.2, 0.7)	0.07

MQSGA, modified quantitative subjective global assessment. SF-36, Short Form-36 health survey.

^a^ Dialysis modality, hemodialysis was taken as a reference (*β *=* *0).

Adjusted I, model adjusted for age, gender and body mass index (BMI); Adjusted II, model adjusted for age, gender, BMI, total calorie, protein, carbohydrate and fat consumptions.

Observing the adverse effects of nutrition status on quality of life was unsurprising. As is shown in Table 2, the MQSGA score was inversely correlated with SF-36 (*β* = −16.2, *p* < 0.001). The result remained significant after adjusting for age, gender, BMI, total calorie, protein, carbohydrate, and fat consumption (*β* = −18.1, *p* < 0.001). Interestingly, this association was observed in different age subgroups and patients receiving HD (*β* = −17.4, *p* < 0.001) but became insignificant in patients receiving PD (*β* = −12.3, *p* = 0.07). Of note, though nutritional status did not affect the quality of life in patients receiving PD, it did impact the vitality subscale (*β* = −2.2, 95% CI: −4.0, −0.3, *p* = 0.027) (Table 3).

**Table 3:** Univariate linear regression analysis between nutrient intakes and MQSGA, SF-36 and its subscales in peritoneal dialysis subgroup.

	SF-36	FP	RP	BP	GH	VT	SF	RE	MH	MQSGA
MQSGA	−12.3 (−25.2, 0.7)	−1.6 (−6.3, 3.1)	−1.0 (−2.2, 0.1)	−1.0 (−3.9, 1.8)	−1.8 (−4.0, 0.4)	−2.2 (−4.0, −0.3)	−0.9 (−2.7, 0.8)	−3.4 (−7.5, 0.7)	−0.3 (−1.6, 1.1)	–
Calorie	1.16 (−2.26, 4.58)	−0.03 (−1.36, 1.29)	0.45 (−0.12, 1.02)	−0.20 (−0.99, 0.59)	−0.13 (−0.71, 0.44)	0.26 (−0.23, 0.74)	0.31 (−0.15, 0.77)	0.65 (−0.41, 1.72)	0.02 (−0.33, 0.37)	−0.07 (−0.15, 0.01)
Protein	0.13 (0.00, 0.26)	0.02 (−0.04, 0.07)	0.01 (−0.01, 0.04)	0.01 (−0.02, 0.04)	0.00 (−0.02, 0.03)	0.01 (−0.01, 0.03)	0.02 (0.00, 0.04)	0.04 (0.00, 0.08)	0.01 (−0.00, 0.02)	−0.00 (−0.00, 0.00)
Fat	2.0 (−1.4, 5.3)	0.5 (−0.8, 1.8)	0.5 (−0.0, 1.1)	0.2 (−0.5, 1.0)	−0.1 (−0.7, 0.4)	0.1 (−0.3, 0.6)	0.3 (−0.1, 0.8)	0.8 (−0.2, 1.9)	0.1 (−0.2, 0.4)	−0.0 (−0.1, 0.0)
Carbohydrate	1.0 (−0.0, 2.0)	0.1 (−0.3, 0.5)	0.1 (−0.1, 0.3)	0.0 (−0.2, 0.3)	0.1 (−0.1, 0.2)	0.1 (−0.0, 0.3)	0.1 (0.0, 0.3)	0.3 (−0.0, 0.6)	0.0 (−0.1, 0.1)	−0.0 (−0.0, 0.0)
Fiber	15.8 (0.8, 30.8)	1.5 (−4.5, 7.5)	0.1 (−2.5, 2.8)	1.9 (−1.7, 5.4)	2.3 (−0.2, 4.8)	2.2 (0.0, 4.3)	1.4 (−0.7, 3.5)	3.5 (−1.3, 8.3)	0.0 (−1.5, 1.6)	−0.1 (−0.5, 0.2)
Cholesterol	−0.0 (−0.3, 0.3)	0.0 (−0.1, 0.1)	0.0 (−0.0, 0.1)	−0.0 (−0.1, 0.0)	−0.0 (−0.1, 0.0)	−0.0 (−0.1, 0.0)	0.0 (−0.0, 0.0)	0.0 (−0.1, 0.1)	−0.0 (−0.0, 0.0)	−0.0 (−0.0, −0.0)
Vitamin A	0.1 (0.0, 0.3)	0.0 (−0.0, 0.1)	0.0 (−0.0, 0.0)	0.0 (−0.0, 0.0)	0.02 (0.00, 0.04)	0.02 (0.00, 0.04)	0.0 (−0.0, 0.0)	0.0 (−0.0, 0.1)	0.0 (−0.0, 0.0)	−0.0 (−0.0, 0.0)
Vitamin B	−28.7 (−199.0, 141.6)	−11.7 (−77.2, 53.9)	16.7 (−11.8, 45.1)	11.4 (−27.6, 50.4)	−11.9 (−40.2, 16.3)	−9.2 (−33.5, 15.1)	4.5 (−18.7, 27.7)	−7.8 (−61.4, 45.8)	−3.4 (−20.7, 13.8)	−1.4 (−5.4, 2.5)
Vitamin B2	135.6 (−74.1, 345.3)	12.3 (−70.0, 94.6)	25.0 (−10.4, 60.5)	11.9 (−37.2, 60.9)	20.7 (−14.5, 55.8)	21.6 (−8.3, 51.6)	16.7 (−12.0, 45.4)	45.3 (−20.5, 111.1)	0.2 (−21.5, 21.8)	−5.2 (−9.9, −0.5)
Niacin	−0.7 (−12.2, 10.9)	0.1 (−4.3, 4.5)	1.5 (−0.3, 3.4)	−1.3 (−3.9, 1.3)	−0.9 (−2.8, 1.0)	0.2 (−1.5, 1.8)	0.5 (−1.1, 2.1)	1.3 (−2.3, 4.9)	0.0 (−1.2, 1.2)	−0.2 (−0.5, 0.1)
Vitamin C	0.8 (0.1, 1.4)	0.1 (−0.2, 0.4)	0.0 (−0.1, 0.2)	0.1 (−0.0, 0.3)	0.1 (0.0, 0.2)	0.1 (0.0, 0.2)	0.1 (−0.0, 0.2)	0.1 (−0.1, 0.3)	0.0 (−0.0, 0.1)	−0.0 (−0.0, 0.0)
Vitamin E	5.3 (−7.7, 18.3)	−1.2 (−6.2, 3.9)	1.6 (−0.5, 3.8)	1.0 (−2.0, 4.1)	0.8 (−1.4, 2.9)	0.6 (−1.3, 2.4)	0.8 (−1.0, 2.5)	2.6 (−1.5, 6.6)	0.1 (−1.2, 1.5)	−0.1 (−0.4, 0.2)
Calcium	0.3 (−0.0, 0.6)	0.0 (−0.1, 0.1)	0.0 (−0.0, 0.1)	0.0 (−0.0, 0.1)	0.1 (0.0, 0.1)	0.1 (0.0, 0.1)	0.0 (−0.0, 0.1)	0.0 (−0.1, 0.1)	0.0 (−0.0, 0.1)	−0.0 (−0.0, 0.0)
Phosphate	0.1 (−0.2, 0.4)	0.0 (−0.1, 0.1)	0.0 (−0.0, 0.1)	−0.0 (−0.1, 0.1)	−0.0 (−0.1, 0.0)	0.0 (−0.0, 0.1)	0.0 (−0.0, 0.1)	0.1 (−0.0, 0.1)	0.0 (−0.0, 0.0)	−0.0 (−0.0, 0.0)
Potassium	0.1 (−0.1, 0.2)	−0.0 (−0.1, 0.1)	0.0 (−0.0, 0.0)	0.0 (−0.0, 0.0)	0.0 (−0.0, 0.0)	0.0 (−0.0, 0.0)	0.0 (−0.0, 0.0)	0.0 (−0.0, 0.1)	−0.0 (−0.0, 0.0)	−0.0 (−0.0, 0.0)
Sodium	−0.1 (−0.3, 0.1)	−0.0 (−0.1, 0.0)	0.0 (−0.0, 0.0)	0.0 (−0.0, 0.0)	−0.0 (−0.0, 0.0)	−0.0 (−0.0, 0.0)	−0.0 (−0.0, 0.0)	−0.0 (−0.1, 0.0)	−0.02 (−0.07, 0.03)	−0.0 (−0.0, 0.0)
Magnesium	0.7 (−0.3, 1.6)	0.0 (−0.3, 0.4)	0.1 (−0.1, 0.3)	0.0 (−0.2, 0.2)	0.1 (−0.1, 0.3)	0.1 (0.0, 0.3)	0.1 (−0.1, 0.2)	0.2 (−0.1, 0.5)	0.0 (−0.1, 0.1)	−0.0 (−0.0, 0.0)
Iron	5.6 (−9.7, 20.8)	−1.9 (−7.8, 4.0)	1.5 (−1.1, 4.1)	0.2 (−3.4, 3.7)	0.8 (−1.8, 3.3)	1.1 (−1.1, 3.3)	1.2 (−0.9, 3.2)	2.0 (−2.8, 6.8)	0.2 (−1.4, 1.7)	−0.2 (−0.6, 0.1)
Zinc	12.4 (−11.1, 35.9)	−1.7 (−10.9, 7.4)	3.2 (−0.7, 7.1)	0.6 (−4.9, 6.1)	1.2 (−2.8, 5.1)	2.0 (−1.4, 5.4)	3.0 (−0.1, 6.1)	4.3 (−3.1, 11.7)	0.8 (−1.6, 3.2)	−0.4 (−1.0, 0.1)
Selenium	−0.1 (−5.5, 5.3)	−0.0 (−2.1, 2.1)	0.7 (−0.2, 1.6)	−0.4 (−1.6, 0.8)	−0.6 (−1.5, 0.3)	0.1 (−0.7, 0.9)	0.3 (−0.4, 1.1)	0.6 (−1.1, 2.3)	−0.1 (−0.7, 0.4)	−0.1 (−0.2, 0.0)
Copper	7.7 (−155.6, 171.0)	−39.3 (−101.2, 22.5)	7.3 (−20.4, 35.0)	−4.3 (−41.8, 33.3)	−9.5 (−36.6, 17.6)	3.1 (−20.3, 26.5)	14.0 (−7.9, 35.8)	30.2 (−20.4, 80.7)	−0.3 (−16.8, 16.3)	−2.5 (−6.2, 1.3)
Manganese	14.5 (−44.5, 73.5)	−7.7 (−30.4, 15.0)	4.1 (−5.9, 14.1)	−0.4 (−14.1, 13.2)	0.2 (−9.7, 10.1)	0.9 (−7.5, 9.4)	5.2 (−2.8, 13.1)	9.4 (−9.0, 27.8)	2.3 (−3.7, 8.2)	0.1 (−1.3, 1.5)

Depicted are beta-estimates with 95%-confidence intervals from linear regression analysis.

PF, physical functioning; RP, role-physical functioning; BP, bodily pain; GH, general health; VT, vitality; SF, social functioning; RE, role-emotional functioning; MH, mental health. MQSGA, modified quantitative subjective global assessment. SF-36, Short Form-36 health survey.

### Varying association of nutrient intakes with nutritional status in HD and PD subgroup

We found inconsistent results between HD and PD subgroups when analyzing nutrient intakes and nutritional status. For patients receiving HD, the MQSGA score was negatively correlated with the consumption of carbohydrates, fiber, selenium, copper, and Manganese (*β* = −0.02, *p* = 0.032; *β* = −0.3, *p* = 0.031; *β* = −0.1, *p* = 0.006; *β* = −2.3, *p* = 0.025; *β* = −1.3, *p* = 0.003, respectively) (Table 4). However, no statistically significant association existed between the abovementioned nutrients and the MQSGA score in the PD subgroup. Only vitamin B2 adversely correlated with MQSGA score among patients receiving PD (*β* = −5.2, *p* = 0.037).

**Table 4:** Univariate linear regression analysis between nutrient intakes and MQSGA, SF-36 and its subscales in hemodialysis subgroup.

	SF-36	FP	RP	BP	GH	VT	SF	RE	MH	MQSGA
MQSGA	−17.4 (−26.2, −8.7)	0.6 (−1.1, 2.3)	1.0 (−1.6, 3.5) 0.464	0.6 (−0.9, 2.2)	0.4 (−0.4, 1.3)	0.6 (−0.6, 1.8)	−0.1 (−1.5, 1.4)	−0.1 (−2.6, 2.3)	0.1 (−1.1, 1.3)	–
Calorie	2.19 (0.20, 4.18)	0.00 (−0.38, 0.38)	−0.29 (−0.81, 0.24)	−0.32 (−0.65, 0.01)	−0.16 (−0.37, 0.05)	−0.26 (−0.54, 0.01)	−0.10 (−0.43, 0.23)	−0.05 (−0.56, 0.47)	−0.15 (−0.43, 0.12)	−0.05 (−0.10, 0.00)
Protein	0.10 (−0.00, 0.20)	0.01 (−0.01, 0.03)	−0.02 (−0.04, 0.01)	−0.01 (−0.03, 0.01)	−0.01 (−0.02, 0.00)	−0.01 (−0.03, 0.00)	−0.00 (−0.02, 0.01)	0.00 (−0.03, 0.03)	−0.00 (−0.02, 0.01)	−0.00 (−0.01, 0.00)
Fat	2.4 (0.1, 4.8)	0.3 (−0.2, 0.7)	−0.2 (−0.9, 0.4)	−0.2 (−0.6, 0.2)	−0.1 (−0.4, 0.1)	−0.1 (−0.5, 0.2)	−0.1 (−0.5, 0.3)	−0.1 (−0.7, 0.5)	−0.0 (−0.4, 0.3)	−0.0 (−0.1, 0.1)
Carbohydrate	0.4 (−0.3, 1.0)	0.0 (−0.1, 0.2)	−0.1 (−0.3, 0.1)	−0.0 (−0.1, 0.1)	−0.1 (−0.1, 0.0)	−0.1 (−0.2, 0.0)	−0.0 (−0.1, 0.1)	0.0 (−0.1, 0.2)	−0.0 (−0.1, 0.1) 0	−0.02 (−0.04, 0.00)
Fiber	3.7 (−5.7, 13.2)	−1.8 (−3.6, −0.1)	−2.0 (−4.5, 0.5)	−2.2 (−3.7, −0.6)	−0.5 (−1.5, 0.5)	−0.7 (−2.1, 0.6)	−0.0 (−1.6, 1.5)	−0.3 (−2.8, 2.1)	−0.8 (−2.1, 0.6)	−0.3 (−0.6, −0.0)
Cholesterol	0.0 (−0.1, 0.2)	0.0 (−0.0, 0.0)	−0.0 (−0.1, 0.0)	−0.0 (−0.0, 0.0)	−0.0 (−0.0, 0.0)	−0.0 (−0.0, 0.0)	0.0 (−0.0, 0.0)	−0.0 (−0.0, 0.0) 0.926	0.0 (−0.0, 0.0) 0.453	−0.0 (−0.0, 0.0)
Vitamin A	−0.0 (−0.1, 0.1)	0.0 (−0.0, 0.0)	0.0 (−0.0, 0.0)	0.0 (−0.0, 0.0)	0.0 (−0.0, 0.0)	0.0 (−0.0, 0.0)	0.0 (0.0, 0.0)	0.0 (−0.0, 0.0)	0.0 (−0.0, 0.0)	0.0 (−0.0, 0.0)
Vitamin B	−9.0 (−127.5, 109.6)	9.8 (−12.3, 31.9)	−20.8 (−51.4, 9.8)	−11.5 (−31.2, 8.1)	−2.3 (−14.7, 10.2)	−8.3 (−24.8, 8.1)	−9.8 (−29.0, 9.4)	−11.6 (−41.8, 18.7)	−5.8 (−22.2, 10.6)	−0.1 (−3.4, 3.2)
Vitamin B2	−22.0 (−95.5, 51.4)	−9.3 (−22.8, 4.2)	−12.9 (−31.7, 5.9)	−14.4 (−26.2, −2.7)	−6.5 (−14.1, 1.0)	−6.2 (−16.4, 3.9)	−12.0 (−23.6, −0.5)	−7.6 (−26.3, 11.0)	−1.9 (−12.0, 8.2)	−0.5 (−2.4, 1.5)
Niacin	8.5 (0.4, 16.6)	0.3 (−1.4, 1.9)	−0.8 (−3.1, 1.4)	−0.8 (−2.3, 0.6)	−0.7 (−1.6, 0.2)	−0.9 (−2.2, 0.3)	−0.4 (−1.9, 1.0)	0.6 (−1.7, 2.8)	−0.5 (−1.7, 0.7)	−0.1 (−0.4, 0.1)
Vitamin C	−0.3 (−0.9, 0.2) 0	−0.0 (−0.1, 0.1)	0.0 (−0.1, 0.2)	−0.0 (−0.1, 0.1)	−0.0 (−0.1, 0.1)	0.0 (−0.1, 0.1)	0.0 (−0.1, 0.1)	0.1 (−0.0, 0.2)	0.0 (−0.1, 0.1)	0.0 (−0.0, 0.0)
Vitamin E	6.9 (1.4, 12.4)	−0.3 (−1.4, 0.8)	−0.1 (−1.6, 1.5)	−0.1 (−1.1, 0.8)	−0.2 (−0.8, 0.4)	0.1 (−0.7, 0.9)	0.4 (−0.5, 1.3)	1.1 (−0.4, 2.6)	0.1 (−0.7, 0.9)	−0.1 (−0.3, 0.0)
Calcium	−0.0 (−0.2, 0.2)	−0.0 (−0.0, 0.0)	0.0 (−0.0, 0.0)	−0.0 (−0.0, 0.0)	−0.0 (−0.0, 0.0)	−0.0 (−0.0, 0.0)	−0.0 (−0.0, 0.0)	−0.0 (−0.1, 0.0)	−0.0 (−0.0, 0.0)	−0.0 (−0.0, 0.0)
Phosphate	0.1 (−0.0, 0.3)	−0.0 (−0.0, 0.0)	−0.0 (−0.1, 0.0)	−0.0 (−0.0, 0.0)	−0.0 (−0.0, 0.0)	−0.0 (−0.0, 0.0)	−0.0 (−0.0, 0.0)	−0.0 (−0.0, 0.0)	−0.0 (−0.0, 0.0)	−0.0 (−0.0, 0.0)
Potassium	0.0 (−0.0, 0.1)	−0.0 (−0.0, 0.0)	−0.0 (−0.0, 0.0)	−0.01 (−0.02, −0.0)	−0.0 (−0.0, 0.0)	−0.0 (−0.0, 0.0)	−0.0 (−0.0, 0.0)	0.0 (−0.0, 0.0)	−0.0 (−0.0, 0.0)	−0.0 (−0.0, 0.0)
Sodium	−0.0 (−0.1, 0.1)	−0.0 (−0.0, 0.0)	−0.0 (−0.0, 0.0)	−0.02(−0.03,−0.01)	−0.0 (−0.0, 0.0)	−0.0 (−0.0, 0.0)	−0.0 (−0.0, 0.0)	−0.0 (−0.0, 0.0)	−0.0 (−0.0, 0.0)	−0.0 (−0.0, 0.0)
Magnesium	0.3 (−0.2, 0.8)	−0.0 (−0.1, 0.1)	−0.0 (−0.2, 0.1)	−0.1 (−0.1, 0.0)	−0.0 (−0.1, 0.0)	−0.0 (−0.1, 0.0)	−0.0 (−0.1, 0.1)	−0.0 (−0.1, 0.1)	−0.0 (−0.1, 0.0)	−0.03 (−0.10, 0.05)
Iron	2.9 (−5.5, 11.4)	−0.1 (−1.7, 1.4)	−0.5 (−2.6, 1.7)	−1.1 (−2.4, 0.3)	−0.2 (−1.0, 0.7)	−0.4 (−1.6, 0.7)	0.0 (−1.3, 1.4)	0.2 (−1.9, 2.3)	−0.2 (−1.4, 0.9)	−0.2 (−0.5, −0.0)
Zinc	8.0 (−6.1, 22.2)	1.4 (−1.2, 4.1)	−1.1 (−4.9, 2.6)	−1.6 (−4.0, 0.7)	−0.5 (−2.0, 1.0)	−0.3 (−2.3, 1.7)	0.3 (−2.1, 2.6)	0.2 (−3.5, 3.9)	−0.4 (−2.4, 1.5)	−0.3 (−0.7, 0.1)
Selenium	1.3 (−0.3, 3.0)	−0.1 (−0.4, 0.2)	−0.2 (−0.7, 0.2)	−0.2 (−0.4, 0.1)	−0.1 (−0.3, 0.0)	−0.1 (−0.4, 0.1)	0.0 (−0.2, 0.3)	−0.0 (−0.5, 0.4)	−0.1 (−0.3, 0.2)	−0.1 (−0.1, −0.0)
Copper	−33.0 (−102.0, 35.9)	−9.3 (−23.5, 4.9)	−11.1 (−31.0, 8.8)	−11.1 (−23.6, 1.5)	−3.9 (−12.0, 4.1)	−4.4 (−15.1, 6.3)	−4.7 (−17.2, 7.7)	−0.5 (−20.1, 19.2)	−4.2 (−14.8, 6.4)	−2.3 (−4.4, −0.3)
Manganese	20.4 (−10.0, 50.8)	−0.5 (−6.2, 5.2)	−2.9 (−10.8, 4.9)	−3.7 (−8.7, 1.2) 0.146	−2.4 (−5.6, 0.7)	−2.5 (−6.7, 1.7)	−0.8 (−5.8, 4.1)	−0.6 (−8.4, 7.1)	−2.1 (−6.3, 2.0)	−1.3 (−2.1, −0.5)

Depicted are beta-estimates with 95%-confidence intervals from linear regression analysis.

PF, physical functioning; RP, role-physical functioning; BP, bodily pain; GH, general health; VT, vitality; SF, social functioning; RE, role-emotional functioning; MH, mental health. MQSGA, modified quantitative subjective global assessment. SF-36, Short Form-36 health survey.

### Varying association of nutrient intakes with HRQOL in HD and PD subgroups

The relationship between nutrient intake and quality of life was also inconsistent in HD and PD subgroups. As illustrated in Table 4, increased consumption of total calories, fat, niacin, and vitamin E correlated with a better quality of life in patients receiving HD (*β* = 2.19, *p* = 0.035; *β* = 2.4, *p* = 0.043; *β* = 8.5, *p* = 0.044; *β* = 6.9, *p* = 0.017, respectively). However, the results revealed that fiber, vitamin B2, potassium, and sodium intakes were negatively correlated with bodily pain subscale score (*β* = −2.2, *p* = 0.007; *β* = −14.4, *p* = 0.019; *β* = −0.01, *p* = 0.047; *β* = −0.02, *p* = 0.008, respectively). In addition, increased fiber consumption was associated with lower physical functioning (*β* = −1.8 *p* = 0.045), and a higher intake of vitamin B2 was disadvantageous for social functioning (*β* = −12.0, *p* = 0.045).

**Figure 3:**
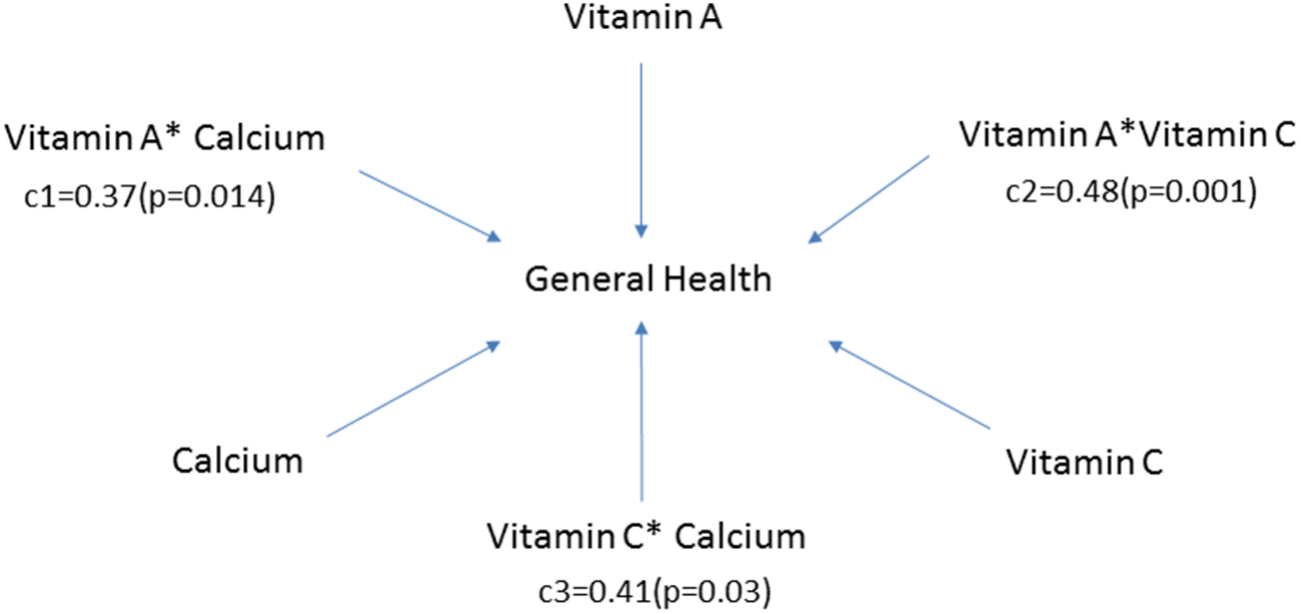
Moderator model for general health subscale in patients receiving peritoneal dialysis. Vitamin A, vitamin C, and calcium were all related to the general health subscale (*β* = 0.02, 95% CI: 0.00–0.04, *p* = 0.028; *β* = 0.1, 95% CI: 0.0–0.2, *p* = 0.015; *β* = 0.1, 95% CI: 0.0–0.1, *p* = 0.006, respectively). In addition, the interaction effects between vitamin A and calcium (c1), vitamin A and vitamin C (c2), vitamin C and calcium (c3) were also significant. c1, c2 and c3 represented the corresponding regression coefficients.

For patients receiving PD, increased vitamin A, vitamin C, and fiber consumption was associated with a better quality of life (*β* = 0.1, *p* = 0.031; *β* = 0.8 *p* = 0.028; *β* = 15.8, *p* = 0.045, respectively). Protein intake positively correlated with social and role-emotional functioning (*β* = 0.02 *p* = 0.0192; *β* = 0.04 *p* = 0.04, respectively). Vitamin A, vitamin C, and calcium intake were positively correlated with the general health (*β* = 0.02, *p* = 0.028; *β* = 0.1, *p* = 0.015; *β* = 0.1, *p* = 0.006, respectively) and vitality subscales (*β* = 0.02, *p* = 0.038; *β* = 0.1, *p* = 0.037; *β* = 0.1, *p* = 0.028, respectively). Furthermore, the interaction effects between vitamin A and calcium (c1 = 0.37, *p* = 0.014), vitamin A and vitamin C (c2 = 0.48, *p* = 0.001), vitamin C and calcium (c3 = 0.41, *p* = 0.03) were all significant for the general health of PD patients. Our results indicated that co-consumption of either of the three nutrients might synergistically promote general health in PD patients (Figure 3).

## Discussion

Following most previous studies (19, 21, 22), we further confirmed that PD patients experienced a better quality of life than HD patients, even in different age groups or nutritional statuses. More importantly, in the current study, we first demonstrated that the effect of nutrient intakes on nutritional status on patients' quality of life varied remarkably in patients receiving different types of dialysis.

Trace elements and most vitamins cannot be synthesized in the body and rely heavily on dietary intake, which are closely related to mitochondrial function and cardiovascular aging. Vitamins B1 and B2 participate in energy metabolism through mitochondrial enzyme coenzymes and maintain the normal functions of blood vessels and the heart (23). Selenium participates in the synthesis of glutathione peroxidase and selenoproteins in mitochondria, plays an essential role in scavenging free radicals and reducing oxidative stress, and contributes to mitochondrial maintenance and cardiovascular health. Selenium deficiencies may lead to impaired mitochondrial function and increase the risk of MACE (24).

Water-soluble vitamin deficiency and trace element disorders may occur during long-term dialysis (25, 26). The water-soluble vitamins with the highest risk of deficiency include vitamin B1 (27), while selenium deficiency among trace elements is constantly observed (26). Our study observed that the daily intake of vitamins B1, B2, and selenium was lower than the dietary intake of healthy people, which is noteworthy for the dialysis population.

Only 25-OH vitamin D is often checked during periodic follow-up examinations of dialysis patients, and deficiencies in other nutrients may only be discovered once obvious complications occur. Therefore, studying the nutritional status of the dialysis population can help reduce nutrition-related complications and cardiovascular aging. Our study has specific clinical significance for improving the quality of life of dialysis patients.

As shown in the current study, patients with PD perceived better feelings in the domains of role-physical functioning, bodily pain, social functioning, role-emotional functioning, and mental health. The fundamental differences between HD and PD can explain these. PD does not require frequent visits to a healthcare center but instead allows patients to perform the procedure at home, giving patients more control over their schedules. Therefore, patients receiving PD maintained social interaction and support more actively and ultimately felt better social functioning and emotional well-being than those undergoing HD. Conversely, HD requires patients to attend dialysis centers three or four times a week for 3–4 h per session, which might negatively affect both personal lives and occupational achievement and thus limit their role in physical functioning (22). In addition, patients on HD were expected to have more problems with pain caused by needle sticks or dialysis access and experience more psychological stress due to being obliged to make more frequent hospital visits and bear a heavier financial burden (28).

Previously, Rambod et al. demonstrated that malnutrition had a negative association with SF-36 (15). A similar result was observed in our study, but only in HD patients and not in PD. The reason for this intriguing observation needs to be clarified. It has been estimated that the prevalence of malnutrition is lower in PD (10%–50%) than in HD (18%–75%) (29). Therefore, the variation in nutritional status might be less evident in patients with PD, thus obscuring the relationship between malnutrition and quality of life. Alternatively, it could be due to the limited number of participants in the PD subgroup, thus diminishing the efficiency of detecting significance. Whatever the reason, malnutrition is a frequent complication associated with an increased risk of mortality and morbidity in dialysis patients and should be paid serious attention to (30). Malnutrition is characterized by a set of mechanisms (31). Besides inadequate nutrient consumption, imbalanced dietary intakes, conditions like inflammation, metabolic acidosis, accelerated catabolism, and endocrine alterations would also contribute to it. Therefore, although intakes of almost all nutrients in the PD subgroup were strikingly fewer than those in the HD subgroup, the malnutrition rate did not differ significantly. We also found that the association between nutrient intakes and nutritional status varied in patients receiving different types of dialysis. HD patients rely more on macronutrients like protein, carbohydrates, and fiber to maintain a normal diet. In contrast, the association between nutrient intake and nutritional status was less evident for PD patients.

The most important finding of this study was that the association between nutrient intake and HRQOL was remarkably different across the two dialytic modes. For patients with HD, it was found that increased consumption of total calories, fat, niacin, and vitamin E was associated with a better quality of life. This result is similar to the finding from the study by Ana Carolina Bonelá dos Santos et al., which showed a positive correlation between consuming calories, protein, fiber, carbohydrates, and quality of life in patients receiving HD (32). However, macronutrients, like protein and carbohydrates, seem less important for patients with PD. Instead, increased consumption of specific micronutrients, like vitamins A and C, correlated with a better quality of life. Interestingly, using the moderation model, our study first discovered that co-consumption of vitamin A and vitamin C might synergistically promote the general health of patients receiving PD.

Nutrient deficiencies, either macronutrients or micronutrients, are commonly seen in dialysis patients due to inadequate consumption and increased loss in the dialysate (2, 33–35). However, it is puzzling why distinctive impacts of specific nutrients were exerted on HRQOL across different dialysis modalities. A possible explanation could be the inherent differences between these two modalities. PD is a more steady, continuous process, while HD is more aggressive and more easily causing hypotension (36). It has been reported that functional disturbance or even ischemic intestine injury could be more severe in HD patients due to a higher risk of hypotension (37). Besides, the more aggressive dialysis schedules with greater hemodynamic instability would be associated with higher gut-derived endotoxin levels (38). In addition, as demonstrated in the study by Betul et al., patients with HD had higher levels of CRP and ferritin compared to those receiving PD (39). All these indicate that intestinal dysbiosis, toxin, or inflammatory status varies significantly across different dialytic modes. This could subsequently influence the absorption, metabolism, or even the *in vivo* bioavailability of certain nutrients, causing a complex association between different dialytic groups. Although these assumptions could not be confirmed in this study, they may provide new insights for future research on individualized nutrient intervention for patients receiving different modes of dialysis.

Although exciting and novel, these results should be interpreted with caution. Firstly, no causal relationship could be determined since this was a cross-sectional study with a limited sample size. We cannot completely exclude the possibility that a better HRQOL in PD patients was caused by their younger age or that patients with a better HRQOL preferred to choose PD *per se*. However, we performed subgroup analyses and adjusted for various confounding factors to obtain a more reliable result. Secondly, due to a lack of serum measurement, the associations of serum levels of certain nutrients and specific facets of HRQOL could not be validated, limiting our findings' generalization.

To our knowledge, this study is the first analysis of sufficient scope to describe the relations of nutrient intakes to nutritional status and specific aspects of quality of life in patients treated with HD or PD. The current study found that PD patients had a better quality of life than HD patients. More importantly, the effects of nutrient intake on nutritional status and quality of life differ remarkably in patients receiving different modes of dialysis. This could provide valuable information and may better understand the extent to which dietary therapy will maintain patients in good nutrition and facilitate tailored nutritional management to enhance patients' quality of life.

## Conclusions

The nutritional intake of PD patients and dialysis patients affects their quality of life differently. Macronutrients significantly impact HRQOL in HD patients, while vitamins have a more substantial impact on PD patients.

## Data Availability

The original contributions presented in the study are included in the article/Supplementary Material, further inquiries can be directed to the corresponding author.
